# Supplementary material for: Clostridium perfringens Epsilon Toxin Binds to and Kills Primary Human Lymphocytes
Source: Toxins (Basel). 2023 Jun 29;15(7):423. doi: 10.3390/toxins15070423 (PMC10467094; doi:10.3390/toxins15070423)
Supplement: Supplementary file 1 [file toxins-15-00423-s001.zip › toxins-2349959-supplementary.pdf]

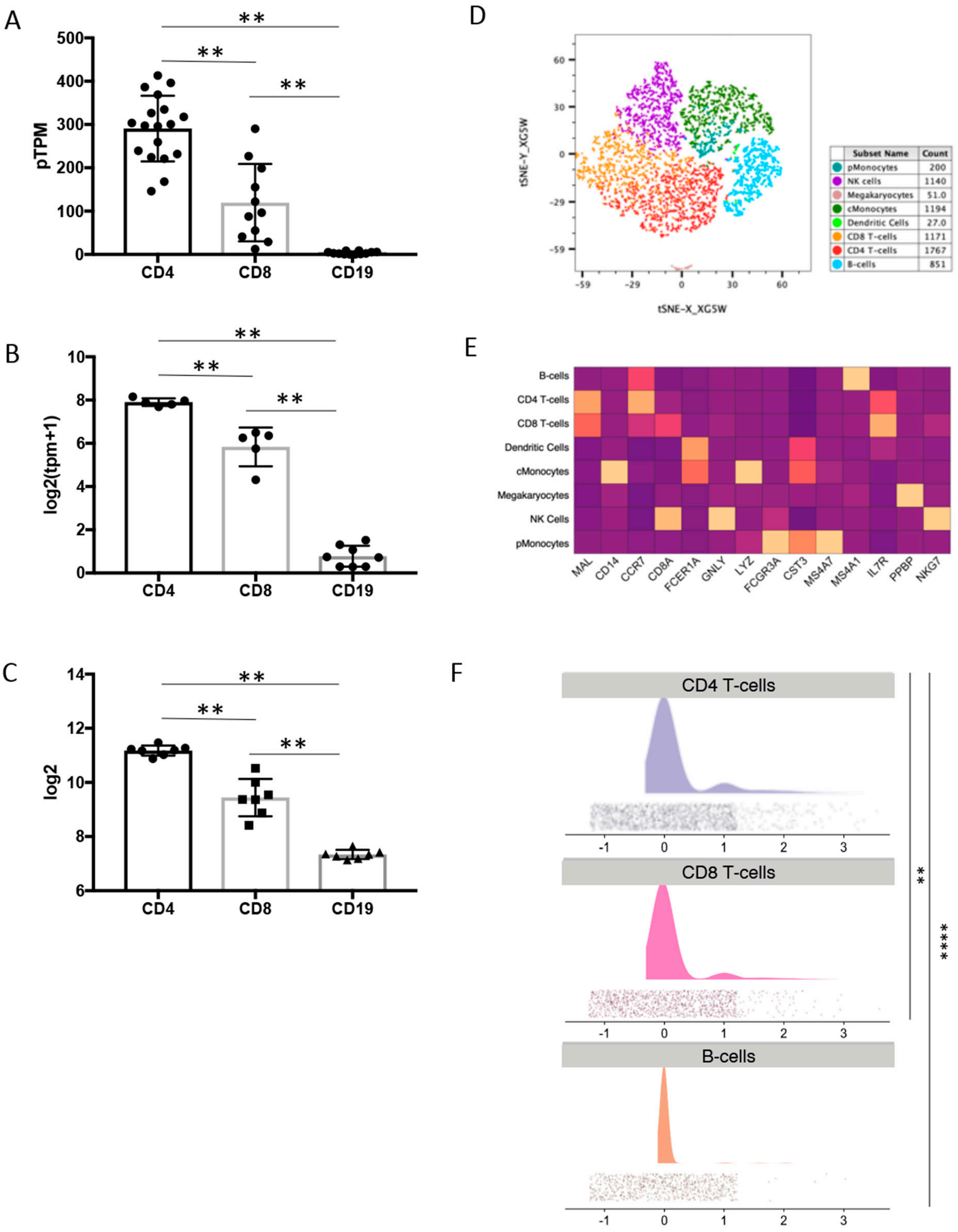

Supplementary Figure S1. Confirmation of *Mal* gene expression in primary human lymphocytes from publicly available datasets. **(A)** *Mal* gene expression in CD4+, CD8+, and CD19+ cells determined via RNAseq analysis. Data was obtained from https://www.proteinatlas.org/ and exported into Microsoft Excel and Prism 7 software. Original publication Uhlen et al [68]. Results are expressed as pTPM and are

from six separate donors. Cells isolated via FACS sorting. CD4 cells include naïve CD4 cells (CD3+, CD4+ CD45RA+), memory CD4 cells (CD3+, CD4+, CD45RA-) and t regs (CD3+, CD4+, CD35+ CD127low, CCR4+, CD25+). CD8 cells include naïve CD8 cells (CD3+ CD4- CD8a+ CD45RA+) and memory CD8 cells (CD3+ CD4- CD8a+ CD45RA-). CD19 cells include naïve B cells (CD3- CD19+ CD27-) and memory B cells (CD3- CD19+ CD27+). ** $p<0.01$ determine by One-way ANOVA with post-hoc Tukey HSD Test. **(B)** MAL gene expression in CD4+, CD8+, and CD19+ cells determined via RNAseq analysis. Data was obtained from https://www.haemosphere.org/ and exported into Microsoft Excel and Prism 7 software. Original publication Choi et al [70]. Results are expressed as Log2(tpm+1) and are from 3 to 5 donors. Cells isolated via FACS sorting. CD4 cells are CD3+ CD19- CD56- CD4+. CD8 cells are CD3+ CD19- CD56- CD8+. CD19 cells include Naïve B cells (CD3- CD19+ CD27-) and memory B cells (CD3- CD19+ CD27+). ** $p<0.01$ determine by One-way ANOVA with post-hoc Tukey HSD Test. **(C)** MAL gene expression in CD4+, CD8+, and CD19+ cells determined via microarray analysis. Data was obtained from https://www.haemosphere.org/ and exported into Microsoft Excel and Prism 7 software. Original publication Watkins et al.[69]. Results expressed as log 2 and are from seven separate donors. CD4+, CD8+, and CD19+ cells were isolated using automated magnetic labeling protocol. ** $p<0.01$ determine by One-way ANOVA with post-hoc Tukey HSD Test. **(D-F)** MAL gene expression in CD4+, CD8+, and CD19+ cells determined via scRNAseq analysis. Five pooled healthy donor PBMNC output files in csv format were exported from https://support.10xgenomics.com/single-cell-gene-expression/datasets (Dataset: 6K_PBMCs from a Healthy Donor, Single Cell Immune Profiling Dataset by Cell Ranger 1.1.0, 10xGenomics). Original publication Zheng et al[67]. Files were imported into SeqGeq v1.6 software (BD Biosciences) for scRNA-Seq data analysis. Quality control was performed in tandem by first eliminating doublets, housekeeping genes and lowly dispersed gene parameters followed by Seurat v3 plugin eliminating genes outside desired expression for phenotyping use. Original publication for Seurat v3 plugin Stuart et al [104]. The data was log normalized and adjusted to a clustering resolution of 0.3 to avoid subpopulations unnecessary for this comparison. Seurat returned eight clustered populations which were characterized and plotted on a t-Distributed Stochastic Neighbor Embedding (t-SNE) graphic **(D)**. The output characterized t-distributed Stochastic Neighbor Embedding (t-SNE) clusters into PBMNC cell types based on previous predictive models. **(E)** Confirmation of unsupervised phenotyping by established marker genes were visualized by mode normalized heatmaps. In addition to canonical phenotype markers, MAL expression is included. **(C)** Lymphocyte clusters (CD4+, CD8+, and B-cells) were then examined for relative MAL expression using the Violin box plugin (https://github.com/TomKellyGenetics/vioplot). Results are presented on a Log2Expression scale evaluated by Mann-Whitney pairwise U-test. **$p<0.01$, **$p<0.0001$ determined by Mann-Whitney pairwise U-test.

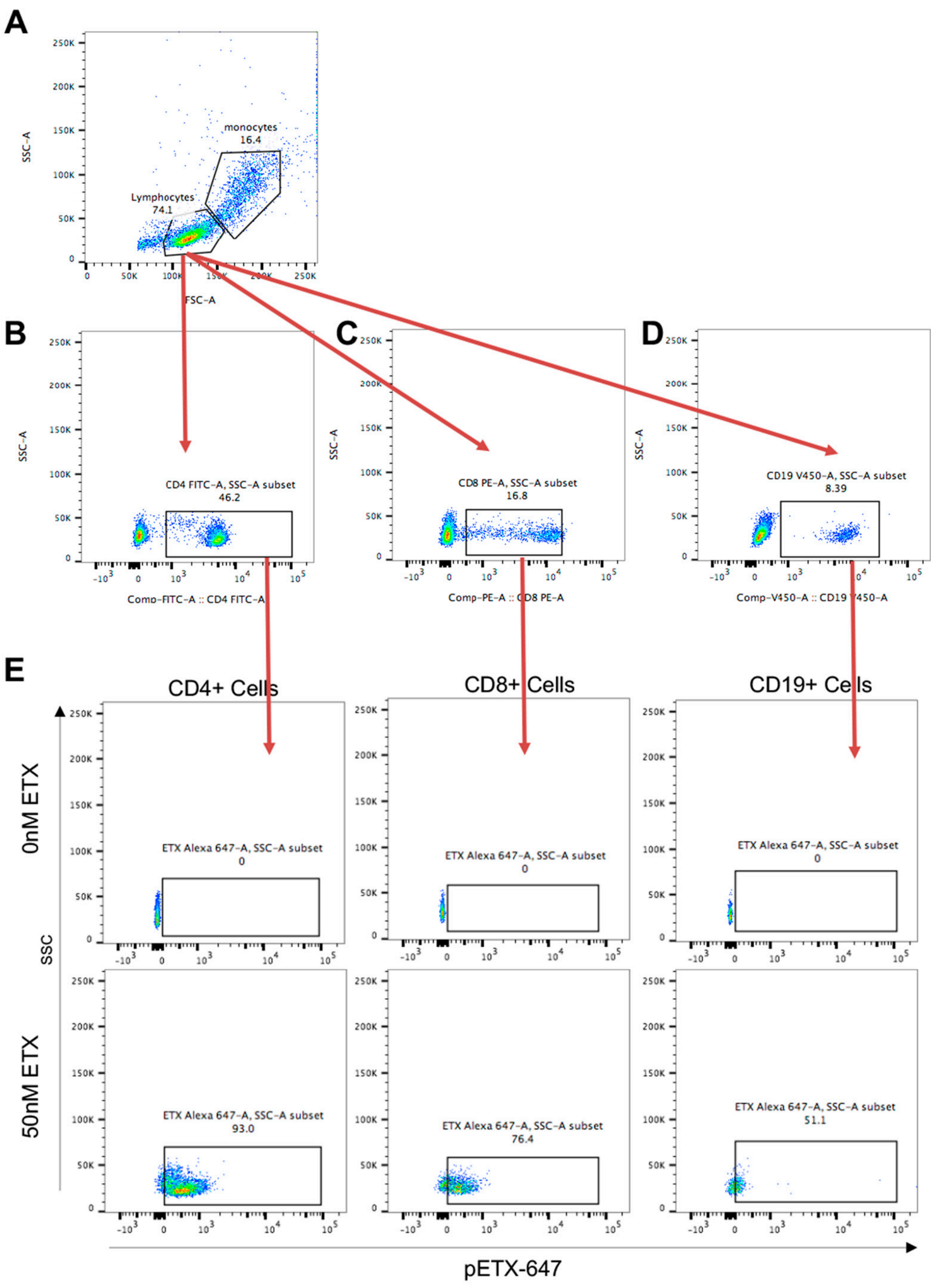

Supplemental Figure S2. Gating strategy for lymphocyte populations. PBMNC were isolated from peripheral blood using density gradient separation. **A)** Lymphocyte and monocyte populations were identified using FSC and SSC profiles. The lymphocyte population was further characterized using cell surface markers to identify CD4+ cells **(B)**, CD8+ cells **(C)**, and CD19+ cells **(D).** Red lines indicate further analysis of gated populations. **(E)** Examples of scatter plots when PBMNC are incubated with 0nM or 50nM pETX-647 for 1 hour. The same data was used in main figure 2a. Results are representative from three separate donors performed in triplicate.

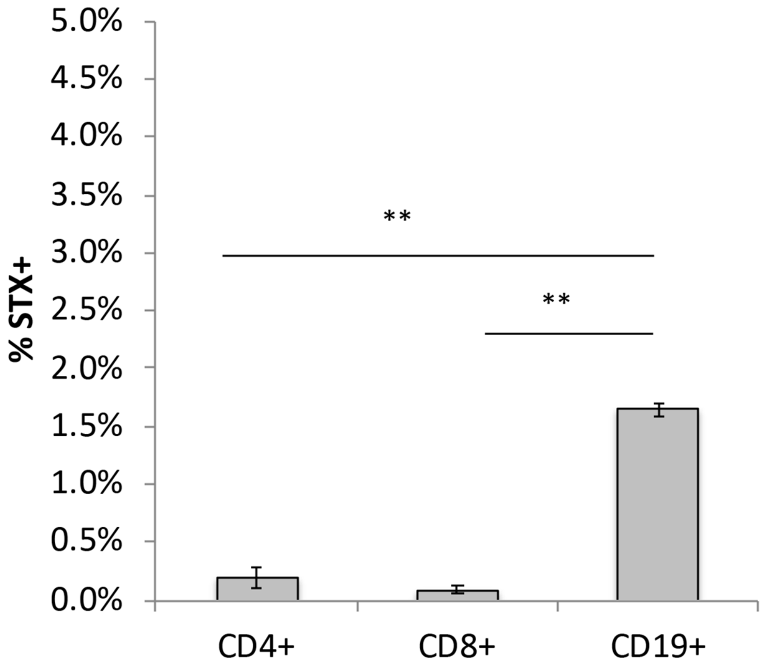

Supplemental Figure S3. STX binding to CD4+, CD8+, and CD19+ cells. To demonstrate pETX-647 binding to human primary lymphocytes is specific and not a result of unspecific binding due to fluorescent labeling, PBMNC were incubated with 50nM of Alexa Fluor 647 labeled Shiga Toxin (STX-647) for 2 hrs at 37°C. STX was labeled with Alexa Fluor 647 Protein Labeling Kit (Life Technologies) as per manufacturer's instructions. Labeled toxin was stored in a 50% glycerol stock (10uM) at -20°C until use. Binding was determined by flow cytometry. Results are expressed as the percent of CD4+, CD8+, or CD19+ cells positive for STX-647 (% STX+). ** $p<0.01$, determine by One-way ANOVA with post-hoc Tukey HSD Test. Results are the mean of one donor performed in quadruplet.

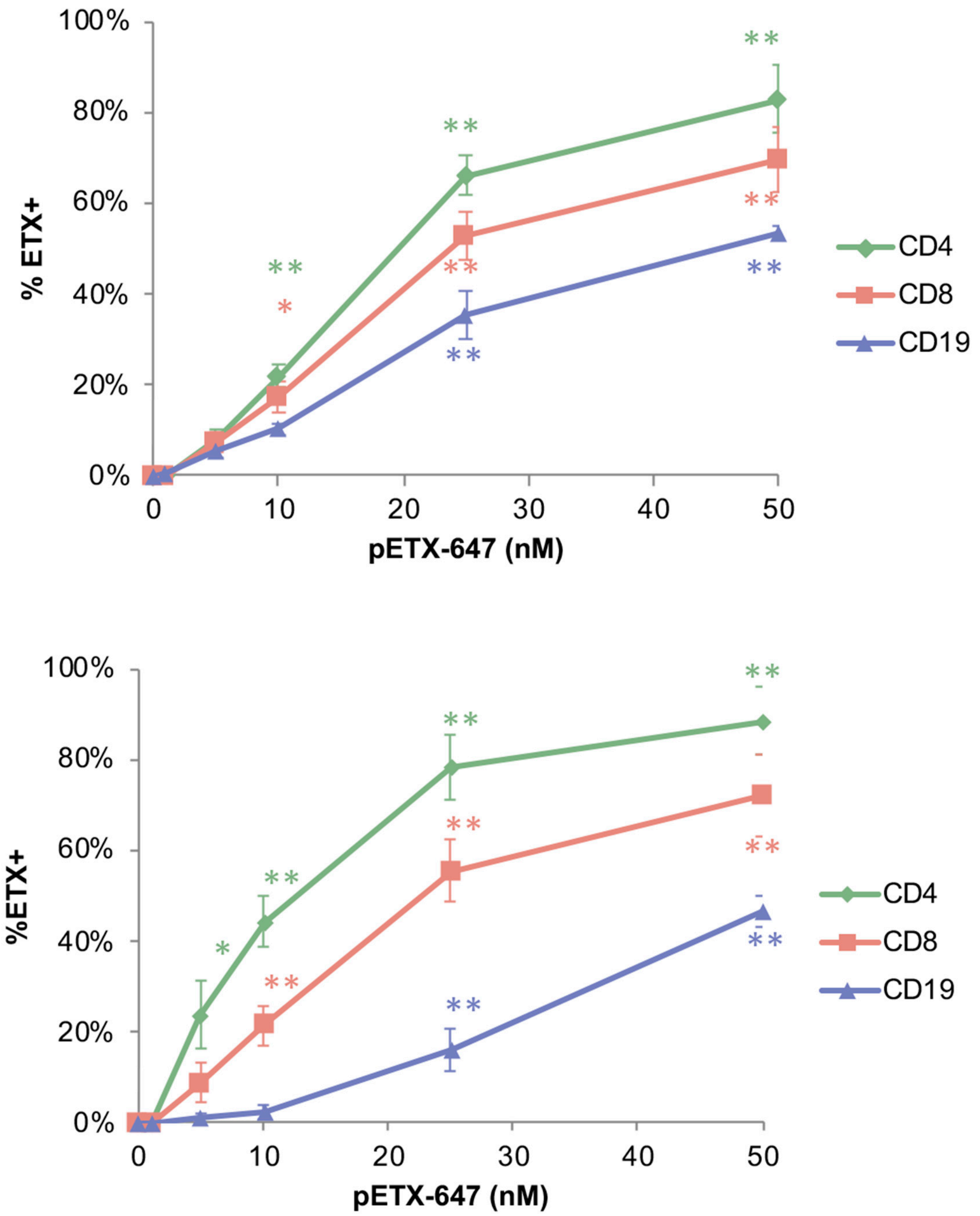

Supplemental Figure S4. Additional time points for dose response evaluation in lymphocyte subsets. To determine if ETX binding was dose dependent, PBMNC were incubated with indicated doses of pETX-647 for 30 and 60 minutes. pETX-647 binding was evaluated by flow cytometry as previously described. Results are expressed as percent ETX positive cells for each lymphocyte population; CD4+, CD8+, and CD19+. * $p<0.05$ and ** $p<0.001$ compared to untreated controls (0nM) as determined by ANOVA. For a more detailed analysis of p values for all pETX doses, refer to supplementary table 1. Results are the mean results of three donors performed in triplicate.

Supplemental Table S1. p values of comparisons for different pETX doses for dose response curves. Performed by ANOVA with post-hoc Tukey HSD Test

COMPARISON			CD4+ Cells				CD8+ Cells				CD19+ Cells			
			15 min	30 min	60 min	120 min	15 min	30 min	60 min	120 min	15 min	30 min	60 min	120 min
0nM	vs	1nM	0.900	0.900	0.900	0.900	0.900	0.900	0.900	0.900	0.900	0.900	0.900	0.900
0nM	vs	5nM	0.807	0.161	0.003	0.001	0.900	0.287	0.378	0.084	0.900	0.133	0.900	0.900
0nM	vs	10nM	0.019	0.001	0.001	0.001	0.439	0.002	0.004	0.001	0.766	0.002	0.846	0.572
0nM	vs	25nM	0.001	0.001	0.001	0.001	0.001	0.001	0.001	0.001	0.001	0.001	0.001	0.001
0nM	vs	50nM	0.001	0.001	0.001	0.001	0.001	0.001	0.001	0.001	0.001	0.001	0.001	0.001
1nM	vs	5nM	0.808	0.161	0.003	0.001	0.900	0.296	0.379	0.082	0.900	0.161	0.900	0.900
1nM	vs	10nM	0.019	0.001	0.001	0.001	0.434	0.002	0.004	0.001	0.778	0.003	0.868	0.588
1nM	vs	25nM	0.001	0.001	0.001	0.001	0.001	0.001	0.001	0.001	0.001	0.001	0.001	0.001
1nM	vs	50nM	0.001	0.001	0.001	0.001	0.001	0.001	0.001	0.001	0.001	0.001	0.001	0.001
5nM	vs	10nM	0.137	0.006	0.008	0.029	0.734	0.084	0.107	0.050	0.900	0.209	0.900	0.900
5nM	vs	25nM	0.001	0.001	0.001	0.001	0.001	0.001	0.001	0.001	0.001	0.001	0.001	0.001
5nM	vs	50nM	0.001	0.001	0.001	0.001	0.001	0.001	0.001	0.001	0.001	0.001	0.001	0.001
10nM	vs	25nM	0.001	0.001	0.001	0.031	0.001	0.001	0.001	0.004	0.002	0.001	0.001	0.001
10nM	vs	50nM	0.001	0.001	0.001	0.004	0.001	0.001	0.001	0.001	0.001	0.001	0.001	0.001
25nM	vs	50nM	0.001	0.001	0.302	0.759	0.001	0.002	0.026	0.270	0.001	0.001	0.001	0.001
